# *Hanseniaspora uvarum* Attracts *Drosophila suzukii* (Diptera: Drosophilidae) With High Specificity

**DOI:** 10.1093/jee/toac029

**Published:** 2022-04-06

**Authors:** Isabella Kleman, Guillermo Rehermann, Charles A Kwadha, Peter Witzgall, Paul G Becher

**Affiliations:** Department of Plant Protection Biology, Unit Chemical Ecology Horticulture, Swedish University of Agricultural Sciences, Alnarp, Box 190, 234 22 Lomma, Sweden; Department of Plant Protection Biology, Unit Chemical Ecology Horticulture, Swedish University of Agricultural Sciences, Alnarp, Box 190, 234 22 Lomma, Sweden; Department of Plant Protection Biology, Unit Chemical Ecology Horticulture, Swedish University of Agricultural Sciences, Alnarp, Box 190, 234 22 Lomma, Sweden; Department of Plant Protection Biology, Unit Chemical Ecology Horticulture, Swedish University of Agricultural Sciences, Alnarp, Box 190, 234 22 Lomma, Sweden; Department of Plant Protection Biology, Unit Chemical Ecology Horticulture, Swedish University of Agricultural Sciences, Alnarp, Box 190, 234 22 Lomma, Sweden

**Keywords:** horticultural pest, invasive pest, pest surveillance, semiochemical, spotted wing drosophila

## Abstract

Since the early phase of the intercontinental dispersal of *Drosophila suzukii* (Matsumura) (Diptera: Drosophilidae), fermentation baits have been used for monitoring. Self-made lures and commercial products are often based on wine and vinegar. From an ecological perspective, the formulation of these baits is expected to target especially vinegar flies associated with overripe fruit, such as *Drosophila melanogaster* (Meigen) (Diptera: Drosophilidae). *Hanseniaspora uvarum* (Niehaus) (Ascomycota: Saccharomyceta) is a yeast closely associated with *D. suzukii* and fruit, and furthermore attractive to the flies. Based on this relation, *H. uvarum* might represent a suitable substrate for the development of lures that are more specific than vinegar and wine. In the field, we therefore, compared *H. uvarum* to a commercial bait that was based on vinegar and wine with respect to the number of trapped *D. suzukii* relative to other drosophilids and arthropods. Trap captures were higher with the commercial bait but specificity for *D. suzukii* was greater with *H. uvarum.* Moreover, *H. uvarum* headspace extracts, as well as a synthetic blend of *H. uvarum* volatiles, were assayed for attraction of *D suzukii* in a wind tunnel and in the field. Headspace extracts and the synthetic blend induced strong upwind flight in the wind tunnel and confirmed attraction to *H. uvarum* volatiles. Furthermore, baited with *H. uvarum* headspace extract and a drowning solution of aqueous acetic acid and ethanol, 74% of field captured arthropods were *D. suzukii*. Our findings suggest that synthetic yeast headspace formulations might advance the development of more selective monitoring traps with reduced by-catch.

Traps baited with attractant lures are a basic and most widely used tool for insect management. The spotted wing drosophila (SWD), *Drosophila suzukii* (Matsumura) (Diptera: Drosophilidae), is a worldwide spreading pest, and trap lures are substantially required for detection and monitoring occurrence and dispersal (Calabria et al. 2012, Walsh et al. 2011, Dalton et al. 2011, Pelton et al. 2016, Kwadha et al. 2021), seasonal population development (Hamby et al. 2014), management decisions (Cha et al. 2018a), finding natural enemies (Abram et al. 2020), and for population control by attract-and kill (Haye et al. 2016, Rice et al. 2017). Development of trap lures has recently been reviewed by Tait et al. (2021).

Due to the relevance and range of trapping applications, a substantial research effort has been made to optimize trap design (Lee et al. 2012, 2013; Renkema et al. 2014; Kirkpatrick et al. 2018) and chemical attractants (Landolt et al. 2012a; Cha et al. 2014, 2017; Kleiber et al. 2014; Frewin et al. 2017; Ðurović et al. 2021; Larson et al. 2021).

The efficacy of insect traps and lures is determined by their attractant power as well as their specificity towards the target species (Wall 1990). Insect pheromones are highly species-specific and efficient at low release rates and therefore widely used for trapping of lepidopteran and coleopteran insects (Witzgall et al. 2010). However, pheromones have so far not been developed for long-range attraction and trapping of *Drosophila* flies (but see Lebreton et al. 2017).

Fermented fruit is known to attract *Drosophila* flies including *Drosophila melanogaster*, and fermentation products like vinegar and wine are therefore applied for fly trapping (Zhu et al. 2003, Becher et al. 2010, Birmingham et al. 2011). Not surprisingly, fermented fruit and vinegar were used also for trapping *D. suzukii*, leading to the development of commercial lures (Kanzawa 1939, Dreves et al. 2009, Landolt et al. 2012a, Cha et al. 2014). For example, one of the commercial traps used for monitoring and mass-trapping of *D. suzukii* is the disposable Riga trap (Riga AG, Ellikon a.d. Thur, Switzerland) which contains a vinegar-wine based bait (Haye et al. 2016). The Riga trap is often used as a reference for comparison with other attractants or traps (e.g. Tonina et al. 2018, Noble et al. 2019, Jones et al. 2021). Wine-vinegar blends were originally studied for the development of *D. suzukii* management by Landolt et al. which led to a monitoring bait based on a mixture of four individual wine and vinegar components forming the backbone of commercially available trap lures (Landolt et al. 2012a,b; Cha et al. 2013, 2015). Despite the wide use of current trap lures, lack of species-specificity has been cited as shortcoming (Cha et al. 2018a, Larson et al. 2021).

From an ecological point of view, vinegar and wine seemingly relate to vinegar flies that infest overripe fruit primarily, whereas *D. suzukii* typically infests fruit even before ripeness (Walsh et al. 2011). Hence, chemical cues more closely related to the ecology of *D. suzukii* could be a basis for developing more specific baits and trap lures (Cloonan et al. 2018).

The yeast *Hanseniaspora uvarum* (Niehaus) (Ascomycota: Saccharomyceta) is associated with *D. suzukii* and found in and on larvae, adult flies, and fruits (Hamby et al. 2012, Bellutti et al. 2018, Lewis et al. 2019). Moreover, previous bioassays demonstrated a strong attraction of *D. suzukii* to *H. uvarum* cultures (Scheidler et al. 2015, Mori et al. 2017, Rehermann et al. 2022). Furthermore, recent work demonstrated attraction of *D. suzukii* to *H. uvarum* in the field emphasizing the predictive value of laboratory studies (Jones et al. 2021). Nevertheless, the potential of *H. uvarum* to improve lure specificity remains to be investigated. We, therefore, compared *H. uvarum* to the Riga bait with respect to the number of trapped *D. suzukii* relative to other drosophilids. In the laboratory, we then tested *H. uvarum* headspace collections and a synthetic blend of selected headspace volatiles in a wind tunnel. Finally, we tested the potential of headspace and the synthetic blend of *H. uvarum* volatiles for *D. suzukii* field trapping.

## Materials and Methods

### Yeast Cultivation and Headspace Sampling

Colonies of *Hanseniaspora uvarum* (CBS 2570; Centraalbureau voor Schimmelcultures, Utrecht, the Netherlands) grown on PDA (BD Difico, Potato Dextrose Agar: 39 g/L) were applied to prepare liquid precultures in PDB (BD Difico, Potato Dextrose Broth: 24 g/L). We used 1 ml of 1-d-old precultures as inoculum of 100 ml PDB to prepare fresh cultures for traps or sampling of headspace volatiles. Cultures (and precultures) were grown on a shaking incubator (25°C, 260 RPM) for 24 h and were in an exponential growth phase. Then, individual yeast cultures were transferred to 500-ml gas wash bottles for collection of the headspace volatiles. Using Teflon tubing, we connected each bottle with a micro gas pump (NMP830KNDC, KNF Neuberger, Inc, NY) that was pushing air through a charcoal air filter into the bottle. A Y-splitter at the gas outlet of the pump allowed to set the air flow (ca. 300 ml/min). The gas outlet of the bottle was connected to a Porapak air filter (Porapak Q, 80/100 mesh, Altech) for trapping the volatiles of the yeast, and further to the gas inlet of the pump. Volatile compounds were collected for four hours and then eluted from each filter with 300 µl ethanol.

### Field Comparison of Riga bait and *H. uvarum* Culture

For all field experiments, we applied Red Drososan traps (Koppert Biological Systems), which are bucket traps that can be used with a liquid attractant in the bottom of the trap in which caught insects drown. In our first experiment, traps were baited with 80 ml fresh culture of *H. uvarum*, or with 80 ml bait transferred from Riga traps, respectively. The yeast cultures were moderately dense (optical density ca. 2.9 at 595 nm) and in exponential growth phase when transferred to the traps, i.e. the medium still contained sufficient resources for *H. uvarum* to survive and metabolize, while being expected to largely outcompete or suppress secondarily introduced microorganisms during the experimental time (Qin et al. 2017).

Traps were distributed on four dates (August 13, August 20, August 29, and September 5 in 2019) at 5 sites on the campus of SLU Alnarp (Sweden), mainly in the landscape laboratory, which is characterized by woodland, shrubs, and field edges, roadside plantations and waterbodies. A trap containing the Riga bait was paired with a trap containing yeast culture at distance of about 5 m. At the first two dates in August, we collected traps after 3 d while on the latter two dates, traps were collected after 4 d. Fruit trees near by the traps were recorded as dogwood (*Cornus* sp.), blackthorn (*Prunus spinosa*), guelder rose (*Viburnum o*pulus), sea buckthorn (*Hippophae rhamnoides*), plums and mirabelle plums (*Prunus domestica*), hawthorn (*Crataegus* sp.), elderberry (*Sambucus nigra*), and morus (*Morus nigra*). Trapped specimen were counted and determined as SWD, other species of the Drosophilidae or other insect or arachnid species.

### Wind Tunnel Tests

A wind tunnel equipped with a piezo electric sprayer was used to measure *D. suzukii* upwind flight attraction to *H. uvarum* headspace or a synthetic mix of headspace components (Becher et al. 2010). The piezo electric sprayer allows controlled vaporization of samples dissolved in organic solvents such as ethanol. The sprayer releases the vapor from a glass capillary horizontally introduced at the upwind end of the wind tunnel. The glass capillary is surrounded by a glass cylinder (60 x 95 mm diameter) which is covered with metal mesh (2 mm pore size) for protection of the set up. When sensing a highly attractive odor flies take off, fly upwind and try to approach the odor source which leads to contact with the metal mesh and most often landing on it.

Yeast headspace extracts were collected from fifteen 100-ml *H. uvarum* cultures cultivated for 24 h in PDB. The Porapak filter eluates of these collections were pooled together and stored in the freezer until used for wind tunnel tests. As highest concentration, we tested *H. uvarum* headspace extract at a concentration in which 1 min of spraying (in volumes of 10 µl/min) corresponded to 2 min of headspace sampling (n = 43 tested flies). In addition, we sprayed a 1:4 (*n* = 56) and a 1:8 (*n* = 39) ethanolic dilution of the extract.

Individual virgin, 3–6 d old, 24 h starved female *D. suzukii* flies from a laboratory rearing (fly stock originating from San Michele all’Adige, Italy) were released at the downwind end of the wind tunnel similar as described earlier (Mori et al. 2017). Fly behavior was observed for 3 min and events of “take-off and upwind flight” as well as “contact” with the metal mesh in front of the odor source was scored.

In addition, we tested fly attraction towards a synthetic blend of seven *H. uvarum* headspace volatiles, which were selected based on their electrophysiological activity on *D. suzukii* antennae (Cha et al. 2012, Revadi et al. 2015, Scheidler et al. 2015, Urbaneja-Bernat et al. 2021). Relative quantities were estimated from GC-MS measurements (6890 GC and 5975 MS; Agilent Technologies; splitless injection onto DB-wax (60 m x 0.25 mm i.d, 0.25 μm film thickness; J&W Scientific, Folsom, CA) with helium as mobile phase at 35 cm/s and a temperature program from 30°C to 225°C at 8°C/min, held for 3 min). Headspace components were tentatively identified based on their Kováts retention indices and mass spectra using the NIST reference library (Agilent), and standard reference compounds. Compounds were quantified based on their peak areas in relation to known quantities of injected reference compounds. The ethyl acetate peak of the ethanolic *H. uvarum* headspace extract, was covered by the ethanol solvent peak, and the ester was therefore quantified from a *H. uvarum* headspace sample that was eluted with hexane (data not shown).

The seven *H. uvarum* volatiles were blended in the following amounts based on the headspace analysis (ng; relative ratio in blend): acetoin (1.48; 7.4), sulcatone (0.02; 0.1), isoamyl acetate (1.16; 5.8), 2-phenylethanol (0.76; 3.8), phenylethyl acetate (0.94; 4.7), ethyl acetate (11.76; 58.8), and isoamyl alcohol (3.82; 19.1) resulting in a total of ca. 20 ng compound per µl ethanol for testing upwind flight attraction (*n* = 40). For getting an understanding of the threshold concentrations for behavioral activity we also tested dilutions of the blend containing 10 ng/µl (*n* = 36) and 1 ng/µl (*n* = 40) total compounds. Fly behavior, when exposed to ethanol, was measured to control the effect of the organic solvent, which was used for preparing the synthetic blends and headspace extracts (*n* = 40). All chemicals were obtained from Sigma Aldrich, but 2-phenylethanol from Merck.

### Field Comparison of *H. uvarum* Headspace Extract, *H. uvarum*-Based Synthetic Blend and a Reference Blend

Based on the results from the wind tunnel assay, we performed a second field experiment at the SLU landscape laboratory, in which we compared catches from Drososan traps that emitted either collected *H. uvarum* headspace extracts or the synthetic blend of *H. uvarum* volatiles. In addition, we tested a synthetic reference blend that we formulated based on the study by (Cha et al. 2013), with modification as described below.

Yeast headspace was collected from six 100-ml cultures of *H. uvarum*, cultivated for 24 h in PDB as described above. The ethanolic filter eluates (300 µl per culture) of these six collections were pooled together and stored in a freezer until use for the field experiment.

The seven components of the synthetic *H. uvarum* blend were dissolved in ethanol in the amounts (µg) and relative ratios as follows: acetoin (7.4), sulcatone (0.1), isoamyl acetate (5.8), 2-phenylethanol (3.8), phenylethyl acetate (4.7), ethyl acetate (58.8), and isoamyl alcohol (19.1) resulting in a total of ca. 100 µg compound per µl ethanol.

A volume of 300 µl of headspace extract or the synthetic blend of *H. uvarum* components was added to 1.2-ml glass vials which served as dispensers. The vials remained without lids and were attached with steel wires inside the traps at the height of the entry holes for the insects (one vial per trap). Preliminary experiments showed that the physical separation of the attractant (dissolved in ethanol and emitted from glass vials fixed at height of the trap openings) in distance to a soapy aqueous drowning solution (in the bottom of the trap) made it difficult to get flies down into the drowning solution. We, therefore, adjusted the set up and applied water 91 ml, tween 0.003 ml, acetic acid 1.6 ml, and ethanol 7.2 ml as drowning solution comparable to the design used by Cha et al. (2013), i.e. vapors from water-soluble acetic acid and ethanol emitted from the drowning solution and merged with the volatiles emitted from the glass vials containing *H. uvarum* headspace extract or the synthetic *H. uvarum* compounds. For reference, using the same drowning solution, we baited traps with methionol as neat compound released from an open glass vial and, in a separate glass vial, acetoin at a concentration of 100 µg/µl ethanol. This reference was formulated based on the bait developed by Cha et al. (2013), however, acetoin was ten times lower concentrated compared to the aqueous acetoin solution used in the original study (Cha et al. 2013), as we had difficulties in dissolving the compound.

Traps baited with the three different treatments (*H. uvarum* headspace extract, synthetic blend of *H. uvarum* volatiles, or the synthetic reference blend) were distributed at two dates (November 14th and 18th, 2019) at three sites of the landscape laboratory. At each site, traps with the three different treatments were placed in a triangular arrangement with ca. 5 m between the traps. Traps were collected after 1 d (November 15th) or 3 d (November 21), respectively, and trap catches were compared based on the number of caught insects per trap and day.

### Data Analysis

Analyses were performed using R statistical software (R Core Team 2020). For analyzing the total number of arthropods, drosophilids, and SWD caught in traps baited with *H. uvarum* or Riga, a generalized linear mixed model (GLMM) with a Poisson error distribution (R software package ‘lme4’) was applied. The specificity of each bait for trapping SWD, either relative to other drosophilids or relative to the total number of trapped arthropod specimen, was analyzed with a GLM fitted with a binomial error distribution for each of the four evaluated trapping periods. For comparison of SWD catches with traps baited with lures that were based on *H. uvarum* headspace extracts, a synthetic blend of *H. uvarum* volatiles, or a synthetic reference blend, a GLMM with a Poisson error distribution was applied followed by a Tukey´s contrast pairwise comparison between the different lures (R software package “multcomp”). The proportion of trapped SWD relative to other drosophilids and to the total of arthropods attracted by the three lures, was analyzed with a GLMM fitted with a binomial error distribution. Sampling dates had no significant effect on the trapping of SWD and data from different dates were therefore combined. The “upwind flight” towards the released volatile stimuli and “contact” with the odor source in the wind tunnel was modeled with a GLM fitted with a binomial error distribution. A Tukey´s contrast test was used for pairwise comparison of fly attraction to different *H. uvarum* headspace dilutions, and for comparison of attraction to different concentrations of the synthetic blend. Residuals were analyzed to verify the distribution of the errors and figures were drawn using “Tidyverse” (R software package “tidyverse”).

## Results

### *Hanseniaspora uvarum* Attracts SWD More Specifically Than a Wine-Vinegar Based Attractant

**Fig. 1.**
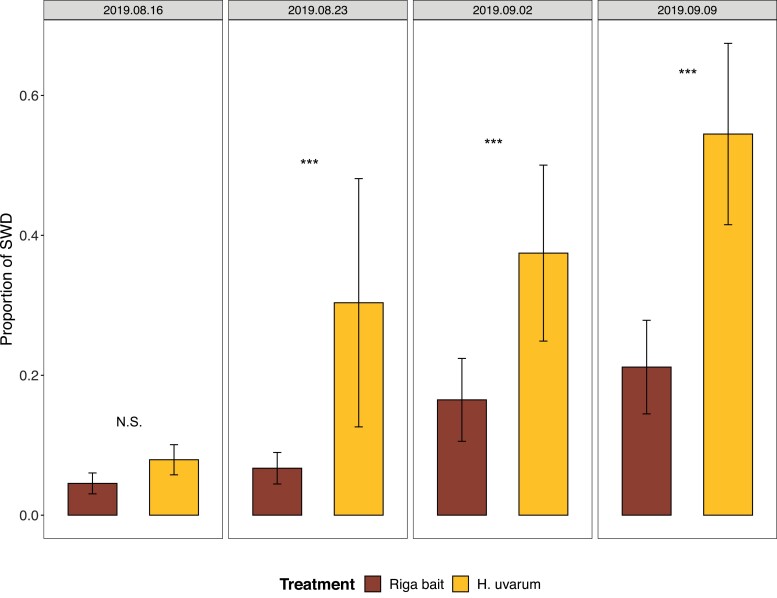
Proportion (Mean ± SEM) of *Drosophila suzukii* (SWD) flies relative to other drosophilids caught with Drososan traps that were baited either with a yeast culture of *Hanseniaspora uvarum* or a vinegar-wine based commercial attractant (Riga bait). The monitoring was performed in four periods between August and September 2019, in a wood and shrub-covered area at Alnarp, Sweden. The dates give the days when traps were collected from the field after 3 d (for the samples 2019.08.16 and 2019.08.23) or 4 d (samples 2019.09.02 and 2019.09.09) exposure. Asterisks indicate significant difference in the proportion of trapped SWD relative to other drosophilid flies caught between treatments (∗∗∗ *P* < 0.001). N.S. indicate no significant difference.

Both *H. uvarum* yeast culture and the Riga bait attracted SWD as well as other drosophilids and arthropods (a few arachnids were trapped, in addition to insects) in a four-week experiment at Alnarp, Sweden. Drososan traps with Riga bait captured significantly more SWD (712 *vs* 445; GLMM Poisson, Z = –8.67, *P* < 0.0001), other drosophilids (4022 *vs* 596; GLMM Poisson, Z = –43.5, *P* < 0.0001) and arthropods (6790 *vs* 1773; GLMM Poisson, Z = –52.38, *P* < 0.0001) compared to yeast culture. During the trapping experiment, from August to September, we saw an increase in the number of total drosophilids that were trapped with the Riga bait (Table 1). Moreover, the proportion of SWD relative to the number of all other trapped drosophilid flies was significantly higher in traps baited with *H. uvarum* during three of the four trapping periods, while no difference was seen during the first period (Fig. 1, GLM binomial: Date 2019.08.16, χ^2^ = 2.73, *d.f.* = 5, *P* = 0.098; Date 2019.08.23, χ^2^ = 14.98, *d.f.* = 8, *P* < 0.001; Date 2019.09.02, χ^2^ = 58.64, *d.f.* = 8, *P* < 0.0001; Date 2019.09.09, χ^2^ = 214.43, *d.f.* = 8, *P* < 0.0001). During the last of the four trapping periods, *H. uvarum* trapped 1.9 times more SWD than all other drosophilid flies together, while on the other hand, traps baited with Riga caught 4.3 times more other drosophilids than SWD.

**Table 1.** Monitoring for *Drosophila suzukii* (SWD) using Drososan traps baited with *Hanseniaspora uvarum* culture or a vinegar-wine based commercial bait (Riga)

Date when trap was collected	Treatment	SWD		Other drosophilids		Arthropods (including all drosophilids)	
		total	per trap	total	per trap	total	per trap
16.08.2019^a^	Riga bait^c^	20	2.2	505	56.1	889	98.8
	*H. uvarum* ^d^	24	2.0	264	22.0	381	31.8
23.08.2019^a^	Riga bait^e^	36	2,4	525	35.0	1,039	69.3
	*H. uvarum* ^e^	16	1.1	54	3.6	169	11.3
02.09.2019^b^	Riga bait^e^	182	9.1	940	47.0	1,574	78.7
	*H. uvarum* ^e^	93	4.7	114	5.7	505	25.3
09.09.2019^b^	Riga bait^e^	474	23.7	2052	102.6	3,288	164.4
	*H. uvarum* ^e^	312	15.6	164	8.2	718	35.9

Shown are the total numbers of trapped SWD, other drosophilid specimen, and arthropod specimen for four different trapping periods, as well as the average number of daily catches per trap.

traps were collected after 3 d in the field;

traps were collected after 4 d;

*n* = 3;

*n* = 4;

*n* = 5

### Headspace Extracts of *H. uvarum* and a Synthetic Blend of Headspace Volatiles Induce SWD Upwind Flight Attraction

**Fig. 2.**
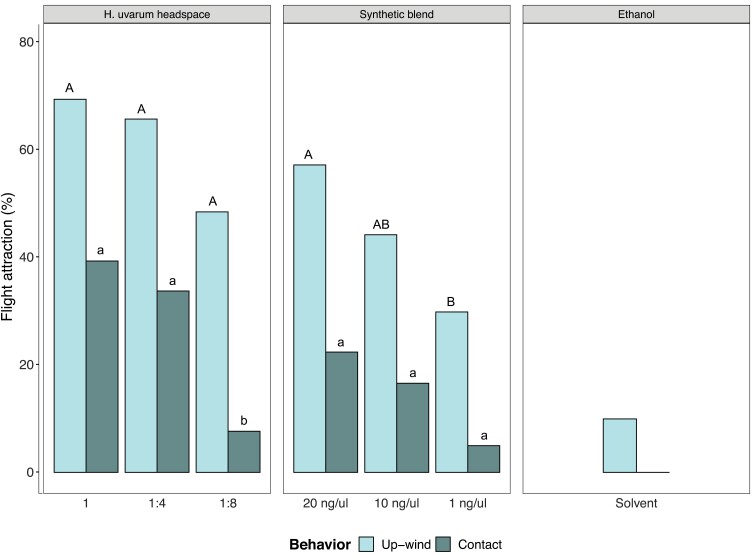
Upwind flight behavior and contact with the odor source of virgin *Drosophila suzukii* females towards vaporized *Hanseniaspora uvarum* headspace extract, a synthetic blend of seven *H. uvarum* volatiles, and to ethanol. In addition to undiluted *H. uvarum* headspace the extract was tested in dilutions of 1:4 and 1:8. The synthetic blend of *H. uvarum* volatiles was evaluated at three concentrations dissolved in ethanol: 20 ng/µl, 10 ng/µl, and 1 ng/µl. Vaporized *H. uvarum* headspace extract induced strong upwind flight attraction, even at 1:4 and 1:8 dilution. Contact with the odor source was reduced at the highest dilution. Upwind flight to the synthetic headspace blend was highest at 20 ng/µl and decreased significantly at 1 ng/µl. Only few flies exposed to ethanol showed upwind flight, but no contact. Different letters denote significant difference between *H. uvarum* headspace dilutions or the synthetic blend concentrations (*P* < 0.05, uppercase for upwind behavior, lowercase for contact behavior).

Wind tunnel experiments were performed to test the attraction of SWD to *H. uvarum* headspace samples or a synthetic blend of headspace volatiles during a 3 min test period. Samples were dissolved in ethanol and evaporated at the upwind end of the tunnel. Control experiments showed that only few SWD took upwind flight towards ethanol vapor while none of the flies was getting close or in contact with the odor source. When *H. uvarum* headspace was vaporized, most flies took off and flew upwind (Fig. 2). Even a 1:4 and 1:8 dilution of headspace extract induced upwind flight while contact with the odor source was reduced at the highest dilution (Fig. 2, GLM binomial distribution, Multiple Comparison of Means (MCM): Upwind flight, *P* > 0.05; Contact, 1 *vs* 1:4, Z = –0.574, *P* = 0.830, 1 *vs* 1:8, Z = 3.043, *P* = 0.006, 1:4 *vs* 1:8, Z = 2.739, *P* = 0.016). At the highest headspace dose, about 40% (17 out of 43 individuals) of the flies contacted the odor source. When the synthetic blends were tested, again a high number of flies took off for upwind flight. The highest dose of the synthetic blend triggered more upwind flight than a 20-times diluted blend (Fig. 2, GLM binomial distribution, (MCM): Upwind flight, 20 ng/ul *vs* 1 ng/ul, Z = 2.443, *P* = 0.039).

### Baits Based on *H. uvarum* Headspace and a Synthetic Blend of Headspace Volatiles Attracted SWD in the Field

**Fig. 3.**
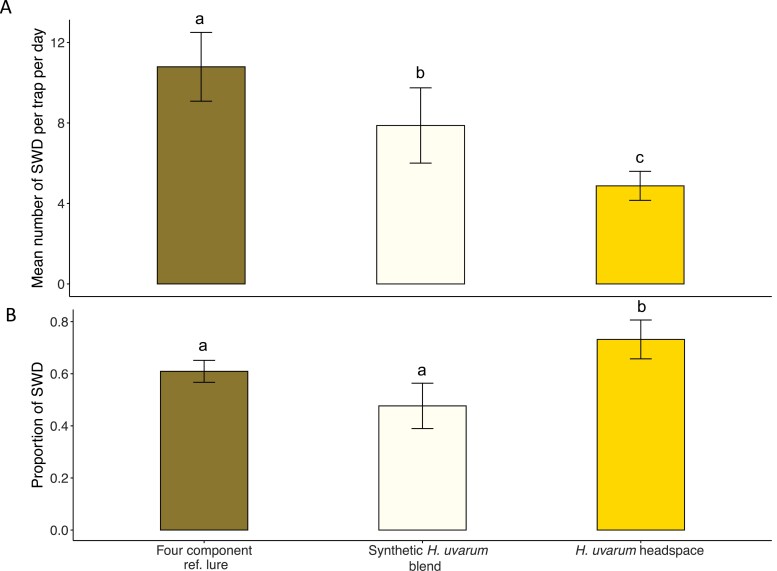
Field trapping with lures based on *Hanseniaspora uvarum* headspace, a synthetic blend of *H. uvarum* volatiles (Synthetic *H. uvarum* blend), and a four-component reference lure. The assay was performed in November 2019, in a wood and shrub-covered area at Alnarp, Sweden (*n* = 6). (A) Mean number (± SEM) of trapped *Drosophila suzukii* (SWD) per trap per day in Drososan traps that were baited with the three different lures. Traps baited with the reference lure caught significantly more SWD compared to the synthetic blend of *H. uvarum* volatiles or the *H. uvarum* headspace extract. (B) Proportion (Mean ± SEM) of SWD relative to other trapped arthropods for each tested lure. While traps baited with the synthetic blend of *H. uvarum* volatiles caught a similar proportion of SWD as the reference lure, traps baited with *H. uvarum* headspace extract showed a higher specificity for attracting SWD. Different letters indicate significant difference between treatments.

Traps baited with *H. uvarum* headspace extract, a synthetic blend of *H. uvarum* volatiles or a synthetic reference blend attracted SWD as well as other drosophilids and arthropods. Overall, the reference blend attracted the highest number of SWD per day (Fig. 3A). Fewer SWD were attracted by the synthetic blend of *H. uvarum* volatiles, and lowest was the average number of SWD in the traps baited with *H. uvarum* headspace extract (Fig. 3A, GLMM Poisson (MCM): *H. uvarum* headspace extract *vs* synthetic blend of *H. uvarum* volatiles, Z = -4.09 *P* < 0.001; *H. uvarum* headspace extract *vs* synthetic reference blend, Z = –7.14 *P* < 0.001; synthetic blend of *H. uvarum* volatiles *vs* synthetic reference blend, Z = 3.365, *P* = 0.002).

However, in comparison to the reference blend or the traps baited with the synthetic blend of *H. uvarum* volatiles, the traps baited with *H. uvarum* headspace extract were significantly more specific in attracting SWD relative to other drosophilids (GLMM binomial (MCM): *H. uvarum* headspace extract *vs* synthetic blend of *H. uvarum* volatiles, Z = 4.36 *P* < 0.001; *H. uvarum* headspace extract *vs* synthetic reference blend, Z = 2.82, *P* = 0.013) or arthropods (Fig. 3B, GLMM binomial (MCM): *H. uvarum* headspace extract *vs* synthetic blend of *H. uvarum* volatiles, Z = 3.88, *P* < 0.001; *H. uvarum* headspace extract *vs* synthetic reference blend, Z = 2.55, *P* = 0.028). In addition to 85 SWD specimen in total, the traps baited with the *H. uvarum* headspace extract attracted only 23 other drosophilids, and 7 nondrosophilid arthropod specimen over the 4 d. In comparison, the traps loaded with the synthetic blend of *H. uvarum* volatiles attracted 122 drosophilid flies in addition to the 148 SWD and 275 arthropods in total, while the synthetic reference blend caught 108 non-SWD drosophilids and 336 arthropods in total, of which 212 were SWD.

## Discussion

The yeast *H. uvarum* is a natural attractant of SWD. Trapping experiments in a wood and shrub covered area in Southern Sweden, showed the attraction of SWD to cultures of *H. uvarum* in comparison to the commercially available Riga bait, which is based on a vinegar-wine formulation. In addition, this study illustrates an increase of the fly population during a four weeks study in late summer 2019 and confirms the establishment of SWD in Sweden where the invasive pest had been documented previously (Manduric 2017). Comparison of SWD field attraction to *H. uvarum* culture and Riga bait, showed that a higher number of SWD and arthropods in general was attracted by the Riga bait. Similarly, Jones et al. (2021) selected Riga traps as a reference when testing different yeasts including *H. uvarum* strains for SWD attraction in the field. Although a different trap was used, results of the studies are similar in the sense that the Riga bait attracted more SWD than *H. uvarum*. However, our data show, in addition, that *H. uvarum* attracted a higher ratio of SWD relative to other drosophilids and that *H. uvarum* was a more specific lure for SWD than the Riga bait. Interestingly, higher specificity became evident only when the overall number of trapped SWD and other insects were beginning to increase during the second week of our study. Whether selectivity could be improved by increasing the overall attraction to *H. uvarum* at low SWD population densities remains to be studied. The higher number of SWD attracted to the Riga bait might have been caused by a higher effective attraction radius compared to the yeast culture (Byers et al. 1989). While it might be possible to increase the effective attraction radius by increasing the *H. uvarum* dose (Schlyter et al. 1992), attraction of flies from a distance is not necessarily helpful for monitoring SWD in fruit and berry crops.

Other, site-specific characteristics may have biased the captures with these two baits. Lures are known to differ in their selectivity and relative efficacy to attract SWD, depending on site-specific conditions such as the crop (Cha et al. 2018a). Odor backgrounds influence the detectability of attractants positively or negatively, and will thus modulate the insect response towards olfactory stimuli (Schröder and Hilker 2008). Background odors with a different impact on Riga and *H. uvarum* lures may have accordingly influenced the differential attraction of SWD. Wind tunnel tests have demonstrated how background fruit odors can influence the attraction of SWD to *H. uvarum* (Huang and Gut 2021), while on the other hand a green leaf odorant background did not affect SWD upwind flight towards *H. uvarum* (Rehermann et al. 2022). The modulation of SWD attraction to volatile compounds in bioassays and field has previously been discussed as a function of background odors (Cha et al. 2018b).

Encouraged by the greater specificity of *H. uvarum* lures in the field, we sampled *H. uvarum* headspace for a wind tunnel bioassay. While wind tunnel upwind flight attraction to the same *H. uvarum* strain has been shown earlier (Mori et al. 2017), we now demonstrated that it is possible to extract behavioral active compounds from yeast headspace, and that SWD was attracted to the vaporized extract in a wind tunnel. Moreover, dilutions of *H. uvarum* headspace collections illustrated a dose-dependent relation between the headspace release rate and the induced attraction. Although the upwind flight response was not significantly different, the percentage of flies contacting the odor source significantly decreased at the lowest headspace dose. Likewise, we previously showed a dose-dependent decrease of upwind flight attraction to vinegar headspace samples in *D. melanogaster* (Becher et al. 2010). Furthermore, SWD flies were similarly attracted to a synthetic blend of seven components of the *H. uvarum* headspace as to the complete *H. uvarum* headspace extract, and attraction decreased with dilution of the blend. The attraction to the synthetic blend of *H. uvarum* volatiles supported the practicability of the approach to select antennally active compounds for generating a mimic of a behaviorally active headspace sample (Tasin et al. 2006). However, not all antennally active components are essential, and 4 out of 15 compounds were sufficient to reach similar attraction of SWD as an authentic mixture of wine and vinegar (Cha et al. 2014).

Based on our wind tunnel results, we used the headspace and the synthetic blend of *H. uvarum* volatiles in a field test. Preliminary tests indicated that these baits attracted SWD into the traps, but without getting the flies in touch with the drowning solution, which was separated from the vials containing the attractants. We, therefore, followed the procedure by Cha et al. (2013) and added acetic acid and ethanol to the drowning solution. A synergistic effect of acetic acid and ethanol as part of the drowning solution has been shown previously (Landolt et al. 2012a, Cha et al. 2014). With acetic acid and ethanol in the drowning solution and *H. uvarum* headspace extract, or the synthetic blend of *H. uvarum* volatiles as baits in the upper part of the trap, we attracted and captured SWD. However, the contribution of the volatile emissions from the drowning solution to the trapping efficiency of the tested baits still needs to be quantified. It is noteworthy, that acetic acid, which is a common yeast metabolite and is also released by *H. uvarum* (De Benedictis et al. 2011) may contribute to the attraction of SWD to live *H. uvarum*. However, our wind tunnel tests demonstrated that SWD is strongly attracted to a synthetic blend of *H. uvarum* components also without acetic acid.

Ethanol is a suitable solvent for eluting volatiles from headspace filters and moreover does not interfere with SWD behavior in the wind tunnel. We, therefore, used ethanol as a solvent for testing the *H. uvarum* headspace collections or blend of synthetic *H. uvarum* headspace volatiles. We are aware that emission of ethanol from the lures exceeds the natural ethanol emission of *H. uvarum* cultures.

The combination of acetic acid and ethanol is considered to be a basic SWD attractant, and addition of the co-attractants methionol and acetoin enhances trap captures (Landolt et al. 2012a, b; Cha et al. 2018b). The synthetic reference bait in our study attracted the highest number of SWD per trap and day, despite a ten times lower concentration of acetoin compared to the formulation used by Cha et al. (2013). Considering the reported dose-dependent influence of acetoin on SWD attraction, a higher number of SWD might have been captured with the original formulation (Cha et al. 2013 and 2017).

Scheidler et al. (2015) demonstrated a distinct preference of SWD for *H. uvarum* in a laboratory assay, and the two esters isoamyl and isobutyl acetate induced a higher electrophysiological antennal response in SWD, compared to *D. melanogaster*. By adding isoamyl acetate and isobutyl acetate, Cloonan et al. (2019) investigated the possibility to increase fly attraction and selectivity of SWD to a four-component mixture of acetic acid, ethanol, methionol, and acetoin (Cha et al. 2014). However, attraction or selectivity to the four-component mixture was not improved, neither in the laboratory nor in the field, and the investigation of other compound blends and ecologically relevant odors was suggested. In view of the ecological and behavioral relevance of *H. uvarum*, and a strong attraction in the wind tunnel assay, we tested an alternative set of *H. uvarum* volatiles as well as *H. uvarum* headspace, in combination with a drowning solution containing the basic attractants acetic acid and ethanol.

Traps baited with *H. uvarum* headspace extract showed the highest selectivity for SWD in comparison to the synthetic blend of *H. uvarum* volatiles or the reference bait. Our data, in conjunction with an established behavioral response of SWD to *H. uvarum*, support the idea that volatiles from ecologically relevant substrates are a valuable resources for the development of more specific lures. More research is needed to clarify redundancy of active compounds in *H. uvarum* and to optimize and reduce a synthetic mimic to the most essential compounds. Moreover, the relevance of the relative ratios of *H. uvarum* headspace components and their concentrations remains to be investigated. Considering yeast strain specific differences and variability of emitted yeast metabolites, which also depend on growth conditions (Spitaler et al. 2020), our synthetic *H. uvarum* blend is a first attempt, and unlikely a mimic of the behaviorally active *H. uvarum* odors that SWD encounters in nature.

Even the development of population control tactics including attracticides will benefit from the identification of highly specific SWD attractants (Mori et al. 2017, Noble et al. 2019, Bianchi et al. 2020, Spitaler et al. 2020, Rehermann et al. 2022, Spitaler et al. 2022).

First and foremost, there is an immediate need to provide efficient monitoring strategies to growers (Tait et al. 2021). Traps that are easy to use, cost-efficient and reliable in detecting SWD at low population densities prior to fruit infestation are a key pest management tool, and will help to reduce precautionary insecticide applications to protect high value crops.
